# Some Methods of Extirpating Pulps and Subsequent Treatment

**Published:** 1900-10-15

**Authors:** James B. Locherty


### SOME METHODS OF EXTIRPATING PULPS AND SUBSEQUENT TREATMENT.

BY DR. JAMES B. LOCHERTY.

Read before The New York Institute of Stomatology, April 3, 1900, and reprinted from the *International Dental Journal*.

Several months ago a paper appeared in the *Dental Cosmos* on the uses of arsenous acid, by an advocate, in which he contended that it was "less painful and less dangerous to dental tissues and the general health of the patient than some of the means suggested as substitutes." He also stated that "it is a successful and conservative method, and although it has been abused in some instances through carelessness and ignorance, yet an intelligent application of it is legitimate and scientific practice;" and among other things was a formula for an arsenous acid paste, also the therapeutic actions of the respective drugs employed therein and the satisfactory results obtained; and, in concluding, the writer stated that if, through carelessness of the patient or from other cause, the peridental membrane becomes inflamed, by applying liquid dialyzed iron the toxic effect would cease, as the arsenous acid would form arsenite of iron. While another practitioner, opposed to the use of arsenous acid, stated that the practice of applying poisonous escharotics, such as arsenous acid, should always be avoided, with the appliances and facilities now at the command of every dentist. "The use of such agents is wholly unnecessary, and especially so when it is considered that the most serious results follow their use in some instances, in either the immediate or remote future, and especially in the cases of those highly susceptible to the poisonous influences of arsenous acid or similar agents; specific results in some instances occurring in twenty-four hours. The results, however, may in other cases be delayed for weeks or months before manifesting any action in a marked manner."

The opinions quoted are illustrations of the widely different views which are maintained on this subject, and were we to look at the subject dispassionately, the conclusion would probably be, that in both the medicinal and surgical method there is much to be commended, notwithstanding that the physiological action of any particular agent may be fraught with much danger. In the adoption of a plan for the extirpation of the dental pulp, the first consideration should be the use of that method which will be the more scientific; that is, not only should the manner of application be thoroughly understood, but also certain considerations regarding either the physiological or pathological action of the agent employed in the treatment.

In considering the medicinal method, the principal agent being arsenous acid, a standard work informs us that it is a powerful caustic; an internal dose is a very severe gastro-intestinal irritant, in minute doses a gastric stimulant causing dilatation of the gastric vessels. As to its effect upon circulation, it says, "The rapidity and force of the pulsation is lessened until it finally stops. It is eliminated by the urine, the alimentary canal, the skin, the saliva, the milk, and even the tears; it may be found many years after death in the bodies of those who have taken it in life. Dose, one-sixtieth to one-tenth of a grain. As a drug it certainly in some way alters the condition of the sufferer, and is vaguely called an alterative." Its action, however, may be considered a purely local one when confined to the pulp cavity and used in infinitesimal quantities. It is probably owing to its being a strong irritant that many practitioners believe that its action distends the vascular tissues, which causes the pulp to be destroyed by strangulation. One writer in *Items of Interest*, for May,

1898, says that " most vasomotor nerve-fibres of the sympathetic system having been found to possess constrictor and dilator functions, when injured or acted upon by certain medicinal agents the vasodilator function persists for some time longer than the vasoconstrictor action ; " he suggests that, " may we not find in this fact an explanation of the action of arsenous acid in the destruction of the pulp ? " the first action being the paralyzation of the vasoconstrictor fibrils ; the vasodilator function continuing, the blood-vessels becoming rapidly engorged, the pulp dies from exhaustion." While another theory is that arsenous acid forms a chemical combination with the hæmoglobin of the blood, called arsen-hæmoglobin, and through its toxic power causes structural degeneration of the cellular elements, followed by the death of the tissues as far as the arsenous acid has penetrated and formed arsen-hæmoglobin (*Dental Cosmos*, September, 1899). Notwithstanding our absence of precise knowledge as regards arsenous acid, it has certainly performed its mission in the destruction of the pulp ; but it is owing to its dangerous properties, and also that permanent relief may be given a patient suffering from a congested or inflamed pulp, a relief not always attended, when arsenous acid is applied, until a certain period of time has elapsed—a sufficient period to cause devitalization of pulp—that has caused us to abandon the use of arsenous acid preparations and use the surgical method in its stead, believing it to be a more scientific method.

The employment of cocain, either separately or in combination, is not absolutely without danger. As to its action, we are informed that "it constricts the arterioles, that a 2 to 10 percent solution is sufficient to anæsthetize the sensory nerves, while a larger dose is necessary to similarly affect the motor nerves." " As to its action upon the respiratory center, it first stimulates it, so that the rapidity and depth of respiration are increased ; but soon depression of the center follows, the respiratory movements become feeble and death takes place from asphyxia. There are, however, several antidotes suggested in the use of hydrochlorate of cocain, suprarenal extract (as suggested by Dr. Dawbarn), whisky, or strychnine. In the minute doses and the manner in which it may be applied locally when necessary for the extirpation of the dental pulp, the toxic power of cocain may be reduced to a minimum ; in fact, experience has taught us that, in the exercise of extreme caution in its application, not the slightest fear need be entertained as to any serious conditions likely to arise in its use when extirpating dental pulps, if the following precautionary measures be adopted, which are, that the rubber dam be used in every instance where practicable; conditions existing where it is impossible to adjust the rubber dam, other measures must be employed in order to confine the agent to the tooth or cavity in question and avoid its being absorbed by the mucous membrane, the great point being to exclude all moisture or exudations from the surrounding tissues.

Several methods are employed in applying cocain, such conditions, for instance, as the position of the tooth, location and extent of cavity governing the special method to be adopted. A few typical cases will serve to illustrate.

*Case* I.—Upper right cuspid; condition of mouth wherein it was advisable to extirpate pulp ; applied rubber dam and ligatured it to tooth ; ground off enamel on palatal portion about on direct line with pulp. When reaching dentin used drill (spear point) for short distance, then applied, in this particular instance, vapocain (an etherealized solution of cocain), although a saturated solution of cocain, or even 10 percent solution, would answer the purpose, but we believe it is not readily absorbed. After a few moments the drill was again used until sensitive dentin was reached, when the application was repeated, and with an ordinary amalgam plugger applied pressure to small piece of unvulcanized rubber to the drilled cavity, believing that in so doing the cocain is made to penetrate the dentin more rapidly. After allowing ample time to elapse for the obtunding of the dentin, used cocain on approaching sensitive dentin, again applying solution, thus continuing cautiously until enabled to enter pulp-chamber with practically no more pain than a patient would experience in excavating a slightly sensitive tooth. The condition present upon reaching the pulp seems to be that the cocain has constricted the blood-vessels in it, so that it not infrequently happens that we are able to enter the pulp-cavity without causing any hemorrhage whatever. The hypodermic syringe is then used, and a 10 percent solution of hydrochlorate of cocain, or saturated solution, injected, say a drop or so, and allowed to more completely anæsthetize the pulp. After a few more moments had elapsed, was enabled to enlarge, by means of rose bur, an artificial cavity to pulp; then by aid of Donaldson's nerve-broach, without handle, taken between thumb and forefinger, and by a rotary or rather twisting or screwing motion, was enabled to pass it up alongside of pulp and extract it in a painless manner. The above instance is a typical case, the patient being of a highly nervous temperament, the operation occupying about thirty minutes.

*Case* 2.—An upper left second bicuspid, cavity distal approximal, pulp slightly exposed; applied rubber dam and ligatures to necessary teeth and then adopted the following method: Used crystals of hydrochlorate of cocain to pulp, allowing ample time for absorption, then by means of instrument worked crystals into pulp-chamber and applied a piece of unvulcanized rubber, using pressure to hold it there for a few moments; and was then enabled, after enlarging the pulp-canal, to remove the pulp. It is astonishing with what rapidity a pulp may be extirpated, and at the same time with but little pain, especially if we allow from twenty to thirty minutes for absorption of crystals of cocain.

*Case* 3.—A lower left first molar; owing to exposure, pulp slightly inflamed, in this instance exposed on the mesial portion, near cervical margin of tooth. In this instance did not apply rubber dam, but by means of bibulous papers and varnish was enabled to keep surrounding parts quite free from moisture. Upon removing filling on mesial approximal portion, was enabled, by means of saturated solution of hydrochlorate of cocain, to reach pulp-cavity in a similar manner as referred to in *Case* 1; then applied sulphuric acid C. P. and cocain, equal parts, to pulp, applying pressure by the aid of unvulcanized rubber; the sulphuric acid, having a great affinity for water, seems to disintegrate the pulp after causing some little pain, not sufficient, however, to cause the patient any great discomfort. By thoroughly enlarging the pulp-chamber I was able to remove its contents, and then, after washing out in a manner shortly to be described, applied the above solution to the individual canals, applying pressure in the manner as before described, and removed contents from canals.

*Case* 3 is cited as an instance of the great vitality which some pulps seem to possess, for the tooth had not only troubled the patient for months, but it was also difficult to extirpate—the sulphuric acid C. P. with cocain is a formula that I would not unhesitatingly recommend, for there is a possibility of some pain attending its application; at least, that has been my experience with it, although it will be found excellent for extirpating so-called nerve-stumps or filaments at the end of root-canals; but in *Cases* 1 and 2 the method may be adopted and applied with uniformly excellent results, the duration of time extending from ten minutes to an hour, depending upon existing conditions. Many other methods have been used, such as eucain, nitrous oxide gas, ethylchloride, cataphoric and air-pressure, with various success, although having used cocain as described in the above cases, especially 1 and 2, with results that have been decidedly satisfactory. I am perfectly willing to continue the treatment until a superior method, especially as to time and safety, may be suggested as a substitute. As to the subsequent treatment in instances where the pulp has just been extirpated and the rubber dam is still in position, the pulp-canal is cleansed by forcing warmed distilled water, by the aid of the hypodermic syringe, until the contents are thoroughly removed, then partially wiping the roots. Sulphuric acid, 50 per-cent solution, is applied—*i. e.*, equal parts alcohol and sulphuric acid—and a few drops of oil of cassia, care, however, being exercised so as not to force it beyond the apical foramen. After applying this agent for a few moments the canal is dried by hot air, the absolute alcohol and hydronaphthol are applied, and the canal filled with chloropercha and gutta-percha cones. This is the general treatment, although other methods are sometimes adopted.

The improvement in the treatment of root-canals has been as marked as other methods have along similar lines in matters pertaining to our profession, and all appreciate the fact that it is absolutely essential to surgically cleanse the canal. There have been many excellent instruments devised for that purpose, in the shape of improved highly tempered nerve-broaches and nerve-canal drills, to remove organic matter which might cause trouble at a future time, yet the physical characteristics presenting themselves in the form of tortuous or irregular canals confront us with a condition in which it is not always practicable to thoroughly remove the pulp in its entirety. What is the best method of treatment to adopt? Of course, if we can employ an agent which will chemically change the remaining contents of the canal into a permanent sterile condition, that will no longer be a source of much annoyance. We still have to consider one or two points suggesting themselves, which are the albuminous matter in the dentinal tubuli, the methods used in attempting to place these tubuli in an aseptic condition, the manner in which agents act producing asepsis, and their germicidal power and stability.

An authority some time ago stated that investigation tended to show that the absorption of liquids into the dentinal tubuli was by osmotic action, and that coagulants soluble in water would diffuse through tooth structure, and also that oleaginous non-coagulants also passed through the tooth structure, although slowly in the presence of water in serum albumin; farther on stating that the function of water in the tubuli was necessary as a carrier of medicinal agents.

This point was simply made touching upon what we believe the importance of moisture or water being present in the tubuli, at least, of the root, to assist us in medicating tooth structure, except in instances where air pressure is used. As to the antiseptic properties of agents, it is well known that under different conditions their actions vary greatly, as, for instance, the action of iodoform, in culture mediums, being an indifferent substance, while in diseased condition it manifests strong antiseptic qualities. "The power of antiseptics depends upon the temperature at which they act, the medium in which they are dissolved, the strength of the solution, the time given them to act, and micro-organisms present in the substances to which they are added." This being the case, it behooves us to use such antiseptics in our treatment as will be quick in action, at the same time being permanent or stable, and while I believe we have not yet been able to find an antiseptic with the latter quality, yet we are fortunate in having many at our command to-day, the application of which, if the proper surgical work be performed at the beginning, will, in almost every instance, place the canal in a thoroughly aseptic condition; then by a suitable root-filling, the patient will have a tooth which will cause him in a vast majority of instances no further trouble in this direction.

### DISCUSSION.

DR. E. C. BRIGGS: If the pulp is removed surgically and aseptically, the tooth will be stronger and more serviceable than before. When surgically removed there is no injury to the pericementum or to the nerves running from the root-canals to the pericementum. In other words, the tooth's vital connection with the body is not disturbed; one has only removed the constructive organ, and, construction being complete, it becomes a superfluous tissue. Of course, to make a perfect result, asepsis and complete mechanical stopping of the canals must supplement the removal. The fact that there are failures to do one or both of these things, owing to faulty manipulations or malformations, does not militate against the underlying principle in the case.

On the other hand, it is my belief that the destruction of the pulp with arsenous acid is too often fraught with danger far-reaching in its results. The tooth too often becomes literally a dead tooth, and too often the tissues beyond the tooth are effected, becoming the starting point of abscesses, dentigerous cysts, and the like.

While I do not believe that arsenic is directly the cause of death, I do not think that many cases, fatal and serious, may be due to actinomycosis, which finds its nidus in the necrotic tissue caused by arsenous acid.

But "necessity knows no law," and I, too, use arsenous acid when it seems necessary. It is my aim, however, in all cases to use it only in part, trying to get the pulp from the canals by surgical means before the arsenic has reached that point. Arsenic is a deadly and subtle poison, and the term caustic or escharotic is no description of its insidious and penetrating toxic action.

I use a 20 percent aqueous solution of the hydrochlorate, and with blunt-pointed syringe or plugs of unvulcanized rubber endeavor to throw the solution *around* the pulp, not into it. It is my theory that the cocain solution jackets the pulp, since in all cases when, from too sharp or too small-pointed a syringe, the pulp has been penetrated, the result has been unsatisfactory.

The injection is begun slowly, so that the first pressure against the pulp shall not produce a shock; then, as the solution gradually forces itself around the pulp, the pressure becomes harder, until finally the piston is pressed down as hard as may be. If all has gone well, there has been no pain, and immediately one can bur out the pulp-chamber and with barbed broaches remove the pulp from the root-canals. The roots are then to be dried out and filled immediately. Waiting even half an hour, as I have done where an emergency case has had to be "sandwiched" in, will show that all life has not been taken from the tooth, as the passage of a broach into the root gives as much pain as if the pulp were still there.

The cases of surgical removal of the pulp by means of cocain done in my office by myself and my four associates number in the hundreds, and the testimony of all is that it is one of the greatest comforts of modern dentistry.

I will not weary you with citation of cases, but state that they include the patients who come to you with toothache from exposed pulp, whether from disease or fracture, neuralgia from pulp-stones, and the rare but necessary instances where teeth must be used as supports for bridges. Some cases of single-rooted teeth have been dismissed from the chair with the root filled within a half hour from beginning the operation. There are some cases of failure, a small percent, however, of the whole number treated. In these cases the cocain has little or no effect. Why, I do not know. It may be non-susceptibility to the drug, or the pulp may be adherent to the walls, thereby not allowing the cocain to get in around it.

Dr. H. W. Gillett: It is my habit to pump in 95 percent carbolic acid after removing the pulp, using fine jewelers' broaches or Donaldson cleansers, and, unless prevented by hemorrhage, to fill the canals at the same sitting. In my hands this procedure has resulted in the retention of good color in the pulpless teeth in all cases, and has been equally satisfactory in leading to practically universal good results as regards usefulness and comfort of the teeth treated.

So far as I am able to trace them, the unsatisfactory results in several years of this practice are limited to one case of failure to get the tip of a lower molar pulp and consequent persistence of sensitiveness, and one case of abscess due to the disappearance of the salol root-filling in a tooth so treated.

It frequently happens that there is more or less soreness for a few days after the operation, this being often dependent (apparently) upon a tendency to go too far in the effort to make sure of all the pulp, or sometimes to forcing the cocain solution too hard.

In my hands I have not found the procedure productive of more discomfort at the time in the average case than the older arsenic method, often the reverse, and I have found it so much more certain to result in a permanently useful and satisfactory appearing tooth that I regard it as an essential to the satisfactory practice of our profession. The practical certainty of freedom from objectionable discoloration with this process can not be too strongly brought out, and I feel that it should be insisted upon as the only admissible method of practice for pulp extirpation in, at least, the ten anterior teeth.

For molars there may be room for difference of opinion, but as regards my own practice I adhere to it there also, in spite of the greater difficulties, (with very rare exceptions) because of the better average condition of the teeth as compared with those in which arsenic has been used.

Dr. E. H. Babcock: I have used arsenous acid preparations almost exclusively. Regarding the dangers of injecting cocain into a tooth, it has seemed to me that they would be very slight, as there would be very little chance for absorption through the apical foramen. The essayist spoke of using the aromatic sulphuric acid in cleansing pulp-canals. I would like to ask, Why use the aromatic in preference to the plain sulphuric acid? As I understand it, the action of the sulphuric acid is simply to dissolve the inorganic part of the tooth.

I always use arsenous acid combined with sulphate of morphia, oil of cloves, and sufficient creosote to make a paste. When I desire to devitalize a tooth, I take a piece of cotton about the size of the head of a pin, and after dipping it into 95 percent carbolic, place upon it a few crystals of cocain, which readily dissolve in the carbolic. I then dip this into the paste and apply. The anæsthetic effect of the cocain, lasting from three to five minutes, is supplemented by the action of the carbolic acid, and that, in turn, by the morphia. It has been my experience that the patient has no pain after the first few minutes. The only condition where I have trouble is where the pulp is partially dead. This is probably due to two causes: first, the congested circulation, which prevents absorption, and, secondly, that there is considerable devitalized pulp to penetrate before the medicament can reach the vital portion. These, I believe, are cases which would be particularly adapted for the use of cocain. I do not believe in using a very large quantity of arsenic, nor in leaving it in longer than twenty-four hours, as a rule.

After the first application of arsenic any remaining vitality can be overcome by the use of carbolic and the crystals of cocain.

Regarding the matter of discoloration, I have never considered that the method of devitalization by the dentist had anything to do with it. It is a matter dependent entirely upon proper cleansing and filling of the tooth.

Dr. J. H. Downes: I use arsenic altogether. I have never tried the surgical operation, but, as I never take particular pains to protect the pulp from moisture, I probably should not have been successful. Arsenic applied in small quantities successfully destroys the pulp with very little inconvenience to the patient, and I doubt very much if the surgical method can be accomplished without some pain. I prefer the arsenic.

Dr. C. O. Kimball: My observations convince me that a tooth destroyed by arsenous acid does not discolor as much as a tooth which dies a natural death. My practice has, for a great many years, been to use arsenous acid for devitalization, but about eight or ten years ago I began using cocain, not so much in devitalizing the pulp as for obtunding the gum in the use of little retaining wedges. I now use it continually, and while at first I had a number of disagreeable cases, I have now almost none. I will say this about the use of cocain, that if used in the mouth in small quantities, and not more than a 2 percent solution, being careful not to let the patient swallow any, it can be used without any difficulty whatever. I use it in all sorts of patients. I have recently been using eucain, but I have had a larger number of cases followed by infection in the use of eucain than with the use of cocain, possibly due to the different action of the two drugs on the capillaries, eucain relaxing, while cocain has the opposite effect of contracting them.

As to the extraction of the pulp under cocain, I can speak positively about that, as I have used it many times. For the anterior teeth, where there is a single root, it seems to me that it is far better than any other method. There is no question in my mind but that it can be done with almost no pain. Indeed. I have tried it many times with this result, questioning the patient very carefully in all cases. In cases difficult of access I prefer the arsenous acid method.

DR. B. F. LUCKEY: It does not seem to me necessary to go very deeply into this subject. The subject of arsenic, its use and abuse, is so old it seems almost elementary. Regarding the use of cocain and eucain in the extirpation of pulps, after a careful and conscientious use of these drugs I have gone back to my first love. arsenic. What I am unable to accomplish with arsenic I can not accomplish with cocain, except for rapid extirpation, and here I find the chloride of ethyl serves my purpose better.

Where the cavity is so far under the gum as to render it difficult to apply the arsenic, my practice has been to drill in from the crown and devitalize the tooth in this manner. It is not necessary to go too far the first time. Drill as far as possible and apply the arsenic for a day or two, and then drill a little farther. In this manner it can be accomplished without trouble. The idea that it is absolutely necessary to place the arsenic in the cavity of decay is a mistake. I have fresh in my mind a case of a suit for damages for five thousand dollars. The cavity was on the labial surface of an upper cuspid tooth extending under the gum. Arsenic was used in a very slovenly manner, with a covering of cotton and sandarac varnish. As a result the patient not only lost that tooth, but the first bicuspid as well, and a large portion of the superior maxilla, from necrosis.

DR. L. C. LEROY: I have been very much interested in the paper, especially in that portion touching upon the toxic effects of the arsenic being allayed by the use of the perchloride of iron, having had two or three troublesome cases. In one, occurring in the mouth of a brother dentist, where extreme caution had been exercised, it was impossible for the drug to have exuded except through the tubuli, possibly where the dentin, cementum and enamel approximate, and this I feel convinced it did. The disturbance was allayed by the use of the perchloride of iron. In another case, inflammation became quite extensive, even extending to the next tooth. Perchloride of iron was injected hypodermically. The change in twenty-four hours was remarkable.

The use of the arsenous acid gives us almost universally good results. I have yet to record a case where I have lost a tooth by its use. In the use of cocain, what becomes of the vital tissue which must necessarily be left in the canal? Does it retain its vitality, or does it subsequently become devitalized? As to the hemorrhage after the use of cocain, this is controlled very easily by the use of the peroxide of hydrogen.

Dr. F. Milton Smith: I congratulate myself that I have learned the secret of successful devitalization of the pulp, and am going home to-morrow to try it on a young man. I have been using arsenic ever since a professor in operative dentistry told me a number of years ago that I must never use it under any condition. I have not found it entirely satisfactory, as so many gentlemen have. I have heard a few gentlemen speak to-night of having no pain following its use. Unfortunately many patients in my vicinity have pulps which do not respond favorably to the application of arsenic, and I have lost teeth from the excessive pain caused by it.

Dr. Locherty: I have been very much interested in the remarks of the different gentlemen present in regard to the treatment of the pulp, for and against arsenic, and it bears out my statement that there is a great diversity of opinion in this regard, and probably will be for some time to come. I used arsenic for a number of years, and then determined to try the cocain method. I used it with varied success for a time and then went back to the arsenous acid. I then had a patient who was taken sick and unable to return to the office at the appointed time, and for that reason I abandoned the use of it altogether and commenced the devitalization and extirpation of pulps by the use of cocain. I have continued to use it ever since, and find that I can accomplish just as thorough and more satisfactory results. So far as pain is concerned, the operation can be performed painlessly, provided sufficient time be taken. I will admit that it does take time, but the operation is such an important one that I believe the time spent is just as important and should be paid for the same as time spent in filling with gold, or any other skillful operation. I have found that cocain has been more effective than eucain, although my experience with the latter might have been too limited. Ethyl chloride I have never used. It seems to me that cold applied to the root of a tooth might cause sloughing if applied too long. As regards the preparation of iron to use to counteract the arsenic, the oxide is the best according to chemistry. To check a hemorrhage when extirpating a pulp under cocain, peroxide of hydrogen is very effective. As cocain has the characteristic of contracting the capillaries, there is very little hemorrhage generally in using such a method, for instance, as described in the typical cases in the paper.

Dr. Bogue: A few years ago Dr. Thomas, of Madrid, mixed cotton with arsenic, cloves, morphia and oil of cinnamon, cut his cotton up in sufficiently fine pieces that a little bit could be taken and put anywhere and covered with gutta-percha and wax. This can not ooze. I find it very satisfactory.

There are thirty-two teeth in the mouth with as many different characteristics as there are teeth. While it might be very easy to remove the pulp in the manner referred to in the six front teeth, it seems to me that it would be very difficult to remove it in this manner in some of the molar roots I have taken out pulps by extreme cold in the six front teeth. The most notable case was that of a gentleman who was not in the chair over twenty minutes I enveloped the tooth in cotton and threw chloride of ethyl upon it; then removed the pulp and filled the root cavity.

Regarding the length of time arsenic can be left in a cavity, I think it has been stated by Dr. Westcott that he has left a treatment of arsenic in for several months without trouble. and it has been said to have been left in as long as three years. I think the fact has been pretty well established that as long as any arsenic remains there is preservation from putrefactive decomposition. Arsenical preparations are used in the preservation of the skins of beasts and birds. The gentleman whose patient returned with the treatment of long standing stated that he found the pulp in a "browned" condition.

Dr. W. Zerfing: I have recently been using in an experimental way to anæsthetize pulps the injection of chloroform with the hypodermic syringe, using a very fine needle. By bringing the needle point just to the exposed tissue and immediately forcing a drop of chloroform on to the tissue, at the same instant also forcing the needle point into it, together with additional chloroform. The needle may in this way be forced with little or no pain well into it when, after a few drops of additional chloroform have been injected, the pulp can instantly be extracted without the slightest sensation. It requires no waiting after injecting, and all appliances should previously be prepared, such as the engine with bur to open the pulp chamber freely if necessary, and whatever extractors or broaches are to be used.

I have recently very successfully taken pulps from incisors, cuspids, bicuspids and molars, without regard to the condition of pulps; also where any sensitive tissue has been left in root-canals with the rest of the pulp dead and perhaps removed, if the needle point can be inserted and a few drops of chloroform forced in, the previously sensitive tissue can be extracted without any pain, while previously perhaps the sensitiveness was so great as to make entrance with a broach impossible. I am using this method quite extensively at present, although time has not permitted the gathering of data to be presented herewith.

